# Amniotic Suspension Allograft Modulates Inflammation in a Rat Pain Model of Osteoarthritis

**DOI:** 10.1002/jor.24559

**Published:** 2019-12-19

**Authors:** Kelly A. Kimmerling, Andreas H. Gomoll, Jack Farr, Katie C. Mowry

**Affiliations:** ^1^ Organogenesis Inc. Birmingham Alabama; ^2^ Department of Orthopaedic Surgery Hospital for Special Surgery New York New York; ^3^ Knee Preservation and Cartilage Restoration Center, OrthoIndy Indianapolis Indiana

**Keywords:** amniotic suspension allograft, osteoarthritis, MIA model, inflammation, pain

## Abstract

Osteoarthritis (OA) affects over 301 million adults worldwide. Inflammation is a recognized component of the OA process; two potent pro‐inflammatory cytokines involved in OA are interleukin‐1β and tumor necrosis factor‐α. Placental‐derived tissues and fluids are known to contain anti‐inflammatory and immunomodulatory cytokines and growth factors. The objective of this study was to evaluate the anti‐inflammatory effects of amniotic suspension allograft (ASA) in an in vivo model of OA; we evaluated pain, function, and cytokine levels following ASA treatment in the rat monosodium iodoacetate (MIA) OA pain model. Rats were injected with 2 mg of MIA, which causes pain, cartilage degeneration, and inflammation, followed by treatment with saline, triamcinolone (positive control), or ASA 7 days following disease induction with MIA. Behavioral assays, including gait analysis, mechanical pain threshold, incapacitance, and swelling were evaluated, along with histology and serum and synovial fluid biomarkers. Treatment with ASA resulted in significant improvements in pain threshold, while weight bearing aversion and swelling were significantly decreased. There were no differences between groups in total joint score after histological grading. Serum biomarkers did not show differences, indicating a lack of systemic response; however, synovial fluid levels of IL‐10 were significantly increased in animals treated with ASA. ASA treatment significantly reduced pain, weight‐bearing aversion and swelling. This study provides mechanistic data regarding potential therapeutic effects of ASA in OA and preliminary evidence of the anti‐inflammatory nature of ASA. © 2019 Orthopaedic Research Society. Published by Wiley Periodicals, Inc. J Orthop Res 38:1141‐1149, 2020

Osteoarthritis (OA) is a degenerative disease involving the soft tissues, cartilage, and subchondral bone of joints, afflicting 301 million adults worldwide.^1^ In the United States, 14 million people had symptomatic knee OA in 2016; of these, two million patients were under the age of 45, and an additional six million between 45 and 64 years old.^2^ Between 2005 and 2030, the expected number of primary total knee arthroplasties (TKA) is projected to grow 673%^3^; of these TKA procedures, OA patients represent 94–97% of the TKA population.^4^ With the average life expectancy increasing each year, there is a need for additional, non‐surgical therapeutic approaches to effectively and safely treat knee OA and delay TKA. Patients with knee OA frequently have comorbidities, including obesity (90%), hypertension (40%), depression (30%), and diabetes (15%).^5^ With obesity and related risk factors on the rise, Maiese et al.^6^ predicts that by 2050, over 130 million individuals will be affected by OA. Cost associated with loss of work due to OA is estimated at $100 billion annually,^7^ while the health care expenditures associated with knee OA were over $27 billion annually.^8^

OA is a multi‐faceted disease, with both biological and mechanical factors at play.^9^ Some mechanical factors leading to degradation include malalignment of the lower extremity,^10^ ligament and meniscal tears,^11^, ^12^ or a combination.^13^ Ligament and meniscal tears often have a biologic effect with degradation mediated through the release of pro‐inflammatory cytokines, including interleukin‐1β (IL‐1β) and tumor necrosis factor‐α (TNF‐α), and matrix metalloproteinases (MMPs).^14^, ^15^ Mechanical factors are exacerbated by excessive weight; it has been reported that every 5 kg gain in weight increases knee OA risk by 36%, with higher body mass index (BMI) leading to more severe knee degeneration.^16^ Furthermore, comorbidities including diabetes^17^ and other sources of low‐grade inflammation, such as metabolic syndrome, have been linked to joint degeneration in OA.^18^ The fundamental role of inflammation in the development of OA highlights the importance of better understanding the underlying cellular and molecular pathways to formulate next‐generation treatment strategies.

One potential treatment approach for OA is the use of amniotic tissue; these tissues are known to be inherently anti‐inflammatory. Amniotic tissue is derived from the human placenta, which is composed of two layers (i) the chorion: the thick, outermost layer in contact with the mother and (ii) the amnion: the thin, innermost layer in contact with the fetus.^19^ The membranes contain the amniotic fluid, which itself contains nutrients and a heterogeneous population of cells including amniotic fluid‐derived stem cells.^19^ Historically, amniotic products have been used to treat a variety of burns and wounds^20^ and applications in ophthalmology in the prevention of corneal scarring after Stevens–Johnson syndrome exfoliation^21^; however, there is growing evidence supporting the use of amniotic tissues in sports medicine applications.^22^, ^23^ There are several commercially available placental‐derived formulations, which differ in their composition (chorion, amnion, cells from the amniotic fluid, amniotic fluid or a combination) and processing methodologies (fresh, dehydrated, or cryopreserved). Components and processing techniques greatly affect what is delivered; for example, dehydration of amnion and chorion tissues has been reported to reduce growth factor levels by 51.1 and 55.5%, respectively.^24^ Amniotic membranes have been reported to contain several immune modulating cytokines and proteins of particular interest for OA, including IL‐1 receptor antagonist (IL‐1Ra), IL‐6, and tissue inhibitors of metalloproteinases (TIMPs).^24^ We hypothesized that a human‐derived amniotic suspension allograft (ASA), composed of cells from the amniotic fluid and particulated amniotic membrane, could play a role in attenuating the pro‐inflammatory environment of OA. Of note, amniotic fluid has been reported to consist of a heterogenic cell population containing endothelial, epithelial, fibroblast, and stem cell populations.^25^ To evaluate the effect of ASA in vivo, a well‐established rat pain model of OA was used to assess pain and other behavioral changes as well as cytokine levels in synovial fluid and serum.

## METHODS

### Rat Monosodium Iodoacetate (MIA) Model Experimental Setup

**Figure 1:**
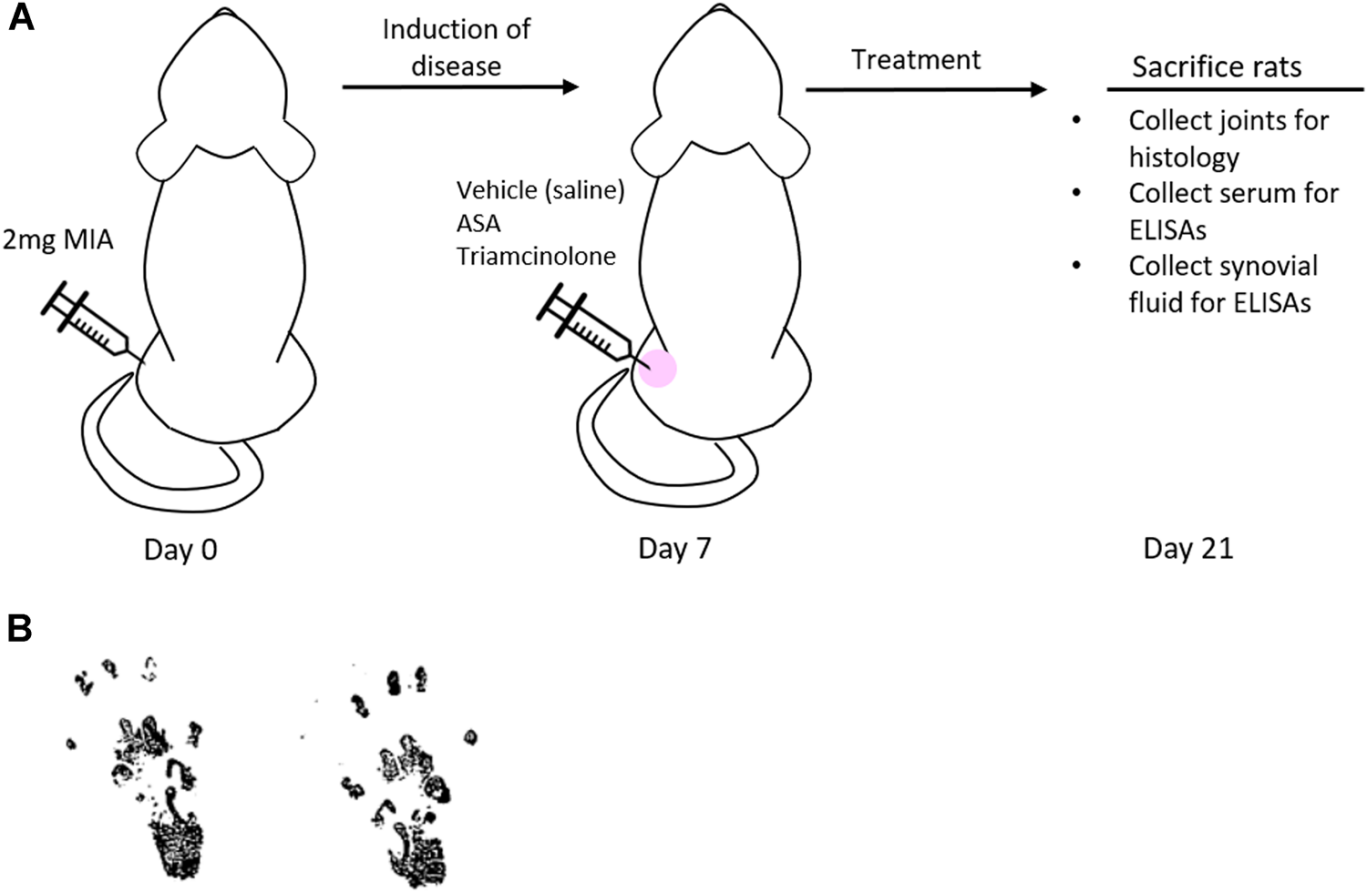
Rat monosodium iodoacetate (MIA) model of osteoarthritis experiment. (A) Study timeline outlining injection of MIA on day 0, treatment of the disease state on day 7, and sacrifice of animals on day 21. (B) Example of footprint pair from gait analysis testing; naïve rat at day 21. [Color figure can be viewed at wileyonlinelibrary.com]

All animal procedures were completed at Bolder BioPATH (Boulder, CO) and protocols were approved by the Bolder BioPATH Institutional Animal Care and Use Committee (IACUC) (Protocol #BBP‐020). Forty‐five male Sprague–Dawley rats (Envigo Harlan, Denver, CO) were obtained and acclimated for 8 days prior to the start of any experiments; animal weights were between 175 and 225 g at the beginning of the study. Animals were housed, 4–5 animals per cage on a 12 h/12 h light/dark cycle; Harlan Teklad diet #8640 was fed ad libitum and unrestricted access to tap water was available throughout the study. On day 0, the right knees of forty rats were injected with 2 mg of MIA to induce OA in the knee (Fig. 1). Five rats did not receive MIA and were reserved as age‐matched controls (naïve group). Following the 7‐day period of hypersensitivity^26^ reported for this MIA model, rats were randomly placed into their treatment group based on incapacitance testing to ensure a balance in disease severity measured by weight‐bearing differences at baseline between all groups. At day 7, rats received an injection into the right knee with one of the following treatments (50 μl volume and *n* = 10 for all groups): saline (vehicle control), 25 μl ASA (25 μl ASA (ASA, ReNu®; Organogenesis, Birmingham, AL) + 25 μl saline), 50 μl ASA (no dilution), or 0.06 mg triamcinolone acetonide injectable suspension (Kenalog®‐10; Bristol Myers Squib, Princeton, NJ; positive control). The volume for dosing was established from a previously reported study with recommendations based on the body weight of rats.^27^ Behavioral testing was carried out at baseline and during the period following treatment; this schedule can be found in Table 1. Other details of the model are shown in Figure 1. Fourteen days after treatment (day 21), rats were sacrificed, and serum, synovial fluid, and knee joints were collected for analysis.

**Table 1:** Behavioral Testing Schedule for Rats

Test	Day 7	Day 8	Day 14	Day 21
Body weight	X		X	X
Incapacitance testing	X	X	X	X
Von Frey analysis	X	X	X	X
Gait analysis	X	X	X	X
Knee caliper	X	X	X	X

Day 7 is the baseline measurement and the day of treatment.

### Behavioral Testing

Rats underwent behavioral testing throughout the study, including incapacitance testing, Von Frey analysis, and gait analysis. In addition, body weight measurements and swelling were evaluated over the course of the study. A testing schedule is available in Table 1. For incapacitance testing and Von Frey analysis, three trials were averaged for each time point evaluated. For gait analysis, rats walked until four clear, evenly inked footprint pairs that represented the gait were obtained. Habituation for incapacitance testing and Von Frey analysis occurred prior to the start of the study, with baseline testing for all assessments occurring on day 7 (after the hypersensitivity period for the MIA induction). Incapacitance testing was completed as previously described in detail.^26^, ^28^ In brief, rats were positioned on a modified incapacitance machine (Stoelting Incapacitance Meter, Wood Dale, IL), and the force exerted by each hindlimb was measured. Von Frey analysis was also completed as previously described.^28^ In brief, filaments of varying diameter were applied to the dorsal surface of the treated hindlimb and the sensitivity was recorded. To analyze gait changes, ink was applied to the ventral surface of both hind limbs and rats walked on paper to generate at least four clear footprint pairs. Gait was then scored based on the footprint of the injured limb compared with the non‐injured limb. Figure 1B demonstrates an example of a footprint pair. Swelling over the course of the study was assessed using digital caliper measurements (Digitrix II micrometer caliper; Fowler & NSK, Newton, MA) of the treated knee.

### Joint Histology

At sacrifice, hind limbs were removed and fixed with 10% neutral buffered formalin (NBF) for 48 h. Following fixation, knees were cut in half in the frontal plane and embedded in the same paraffin block for histological assessment. Sections were stained using standard techniques with toluidine blue for histopathological grading. Whole‐joint graded components are displayed in Table 2; each of these categories were graded on a scale of 0–5, where 0 represents a normal joint and 5 represents severe degeneration.^29^ Total joint score is made up of the sum of the cartilage damage, bone resorption, subchondral bone sclerosis, and osteophyte scores. In addition, joint score is presented as the total joint score minus cartilage; this score is the sum of the bone resorption, subchondral bone sclerosis, and osteophyte scores. Osteophyte measurements were converted to a scaled score, where 0 represents no osteophytes, and five represents a measurement greater or equal to 1,000 μm.

**Table 2:** Histological Scoring

	Naïve	Vehicle	ASA (25 μl)	ASA (50 μl)	Triamcinolone
Total joint score	0.0 ± 0.0	13.5 ± 0.4	13.6 ± 0.4	13.7 ± 0.2	13.1 ± 0.7
Total joint score minus cartilage	0.0 ± 0.0	8.6 ± 0.4	8.6 ± 0.4	8.8 ± 0.2	8.3 ± 0.6
Cartilage damage	0.0 ± 0.0	4.9 ± 0.1	5.0 ± 0.0	4.9 ± 0.1	4.8 ± 0.1
Bone resorption	0.0 ± 0.0	0.95 ± 0.03	0.95 ± 0.03	0.97 ± 0.03	0.98 ± 0.01
Subchondral bone sclerosis	0.0 ± 0.0	0.0 ± 0.0	0.3 ± 0.3	0.0 ± 0.0	1.1 ± 0.5*
Osteophyte measurement (μm)	0.0 ± 0.0	764.0 ± 55.4	696.0 ± 48.5	812.0 ± 35.3	516.0 ± 63.8**
Synovitis	0.0 ± 0.0	2.4 ± 0.2	3.0 ± 0.2	3.3 ± 0.2***	2.9 ± 0.1
Fibrosis	0.0 ± 0.0	2.3 ± 0.2	3.0 ± 0.3	3.9 ± 0.3***	3.0 ± 0.3

Mean ± standard error reported.

* *p* < 0.05,

** *p* < 0.01, and

*** *p* < 0.001 compared with vehicle control by Dunn's post hoc test.

### Serum and Synovial Fluid Assays

At sacrifice, serum and synovial fluid was collected from each rat. Serum was spun down, aliquoted, and placed at −80°C until use. Analysis of cytokines in the serum samples, including IL‐1β, IL‐6, IL‐10, MCP‐1, TIMP‐1, and TNF‐α, were measured using rat enzyme‐linked immunosorbent assay (ELISA) kits (R&D Systems, Minneapolis, MN). All kits were run according to the manufacturer's instructions. Synovial fluid was collected via lavage with 50 μl heparinized saline from the right knee; samples were spun down, aliquoted, and placed at −80°C until use. Analysis of synovial fluid cytokine levels was completed using the Rat Inflammatory Panel Q1 multiplexed sandwich ELISA (RayBiotech, Norcross, GA). This panel included IFN‐γ, IL‐1α, IL‐1β, IL‐2, IL‐4, IL‐6, IL‐10, IL‐13, MCP‐1, and TNF‐α, and was run according to the manufacturer's instructions.

### Statistical Analysis

All statistical analysis was done using GraphPad Prism 6 (GraphPad Software, La Jolla, CA). For animal behavioral and histological data, a one‐way analysis of variance (ANOVA) was run for each time point, with a Dunn's post hoc test to assess statistical significance between groups. For animal serum and synovial fluid data, a one‐way ANOVA was run with a Tukey's post hoc test to assess statistical significance between groups.

## RESULTS

### Rat MIA Behavioral Testing

**Figure 2:**
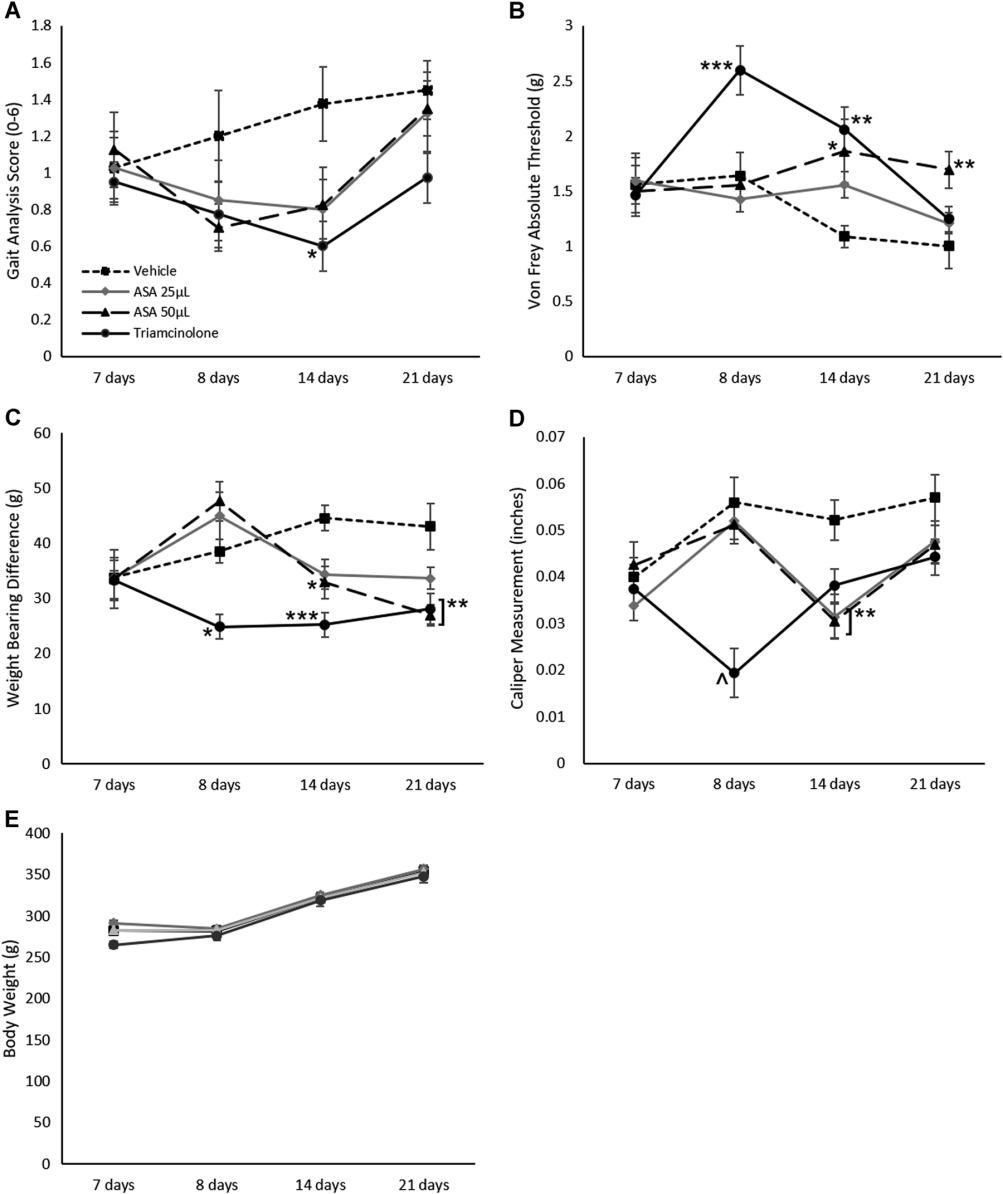
Rat behavioral testing results, including (A) gait analysis, (B) Von Frey analysis, (C) incapacitance testing, (D) knee caliper measurements, and (E) body weight changes. Mean ± standard error reported for each time point; *n* = 10 per group for all figures. *p < 0.05, **p < 0.01, and ***p < 0.001 to the vehicle control by Dunn's post hoc test

The MIA model was chosen to evaluate inflammation and pain in vivo due to its previous validation as a model to evaluate nociception changes.^30^ In this study, treatment was administered seven days following disease induction of OA by an intra‐articular (IA) injection of MIA (Fig. 1A). Body weight measurements, gait analysis, Von Frey analysis, incapacitance testing, and knee caliper measurements were conducted at various points during the study, as shown in Table 1. An example of a footprint pair from the gait analysis is shown in Figure 1B. In dynamic gait analysis, differences between vehicle control and animals treated with ASA increased at both day 8 and 14 but did not reach the statistical significance. Triamcinolone, the positive control, also showed no statistically significant differences at day 8, but a significant improvement in gait analysis score was seen at day 14 (*p* = 0.0207, Fig. 2A).

In addition to sensitivity using dynamic gait, pain was measured by determining pain thresholds via Von Frey assessment. In this study, rats treated with 50 μl of ASA showed a significant improvement in pain threshold levels at day 14 that was sustained until study completion (21 days) compared with vehicle controls (*p* = 0.018 and *p* = 0.0049, respectively; Fig. 2B). Treatment with triamcinolone (positive control) resulted in significant improvement in pain threshold level earlier (day 8) that was sustained until day 14 (*p* = 0.0006 and *p* = 0.0021, respectively; Fig. 2B), but resolved by day 21.

Another behavioral assessment used in this study was incapacitance testing; this testing utilizes weight‐bearing differences between hind limbs to evaluate pain and sensitivity. In this study, treatment of rats with 50 μl of ASA resulted in a significant decrease in weight‐bearing differences between limbs compared with the vehicle control on days 14 and 21 (*p* = 0.033 and *p* = 0.0022, respectively; Fig. 2C). In the positive control group, triamcinolone showed a significant decrease in weight‐bearing difference at days 8, 14, and 21 (*p* = 0.014, *p* = 0.0002, and *p* = 0.0048, respectively; Fig. 2C).

Finally, swelling was assessed through knee caliper measurements of the joint. Administration of ASA resulted in significant decreases in swelling at day 14 compared with vehicle controls (*p* = 0.005 for 25 μl dose, *p* = 0.0031 for 50 μl dose; Fig. 2D). Treatment with triamcinolone resulted in decreased swelling at day 8 only (*p* = 0.0001; Fig. 2D).

To verify that the treatment did not cause any systemic effects, body weight was measured throughout the study. The body weight over the course of the study are shown in Figure 2E. There were no significant body weight changes with ASA or triamcinolone compared with the vehicle control over the course of the study.

### Rat MIA Histology Assessment

**Figure 3:**
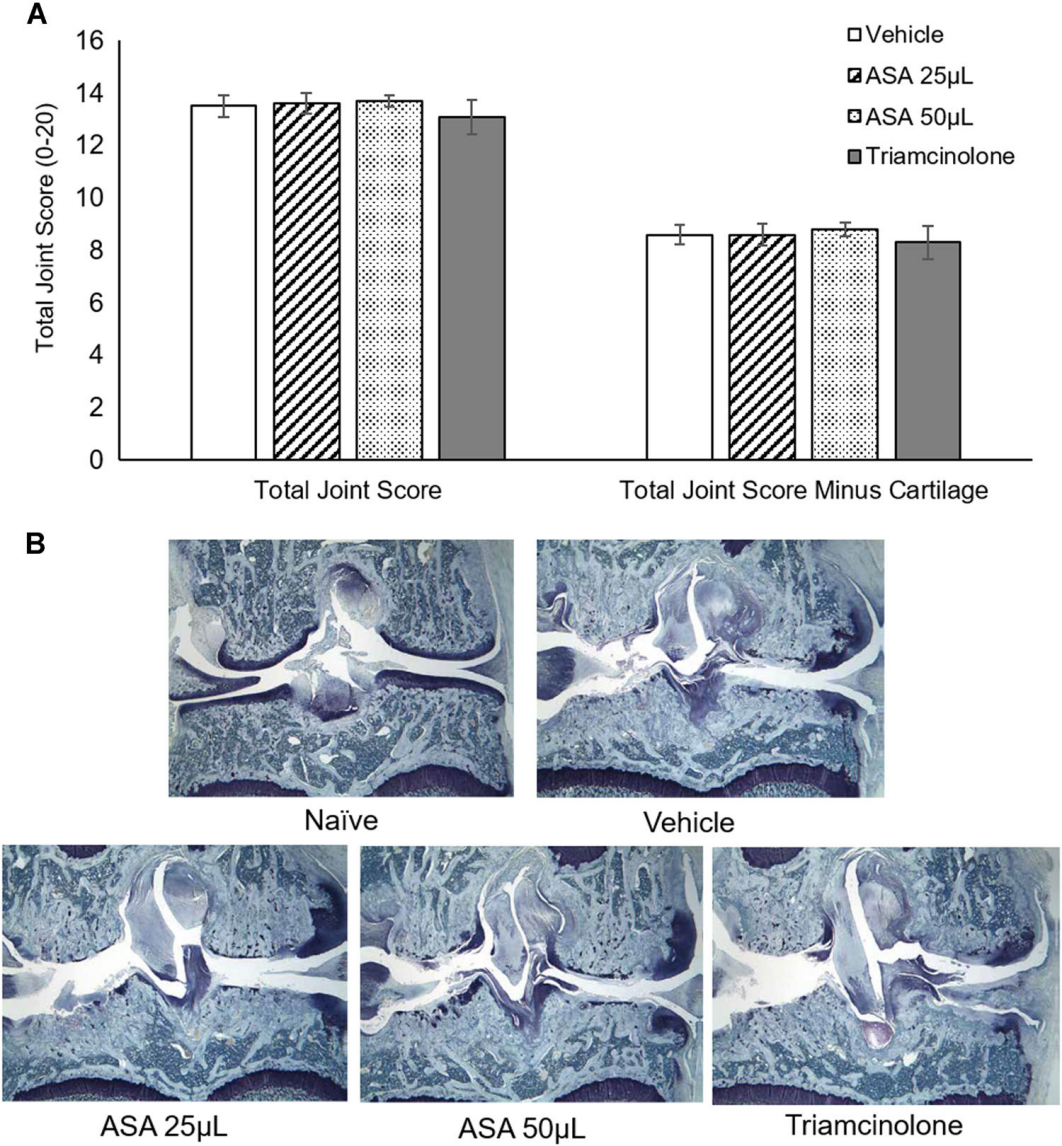
Histology assessment results. (A) Total joint score with and without cartilage damage subscore. Mean ± standard error reported; *n* = 10 per group. (B) Representative images for each group shown. ×16 objective. [Color figure can be viewed at wileyonlinelibrary.com]

The total joint score, which consists of the sum of the cartilage damage, bone resorption, subchondral bone sclerosis, and osteophyte subscores, is shown in Figure 3A. Because the MIA injection inhibits chondrocyte metabolism,^26^ the total joint score was calculated with and without the cartilage damage component. In this study, there were no significant differences with ASA or triamcinolone (positive control) treatment compared with the vehicle control with or without inclusion of the cartilage damage component. Representative images of each group are shown in Figure 3B; qualitative comparisons of the vehicle, ASA and triamcinolone groups to the naïve animals (no MIA injection or treatment) demonstrates the severe widespread cartilage damage associated with the MIA model.

Individual components of the total joint score, along with the synovitis and fibrosis scores, are shown in Table 2. There were no significant differences with any treatments compared with the vehicle control for total joint score, total joint score minus cartilage, cartilage damage, or bone resorption. Triamcinolone had significantly increased subchondral bone sclerosis compared with vehicle control (*p* = 0.0134). In addition, triamcinolone had significantly reduced osteophyte measurements and scores compared with the vehicle control (*p* = 0.0039 and *p* = 0.0018, respectively). Treatment with 50 μl of ASA resulted in a significant increase in synovitis and fibrosis scores compared with the vehicle control (*p* = 0.0008 and *p* = 0.0005, respectively).

### Rat MIA Serum and Synovial Fluid Assessment

**Figure 4:**
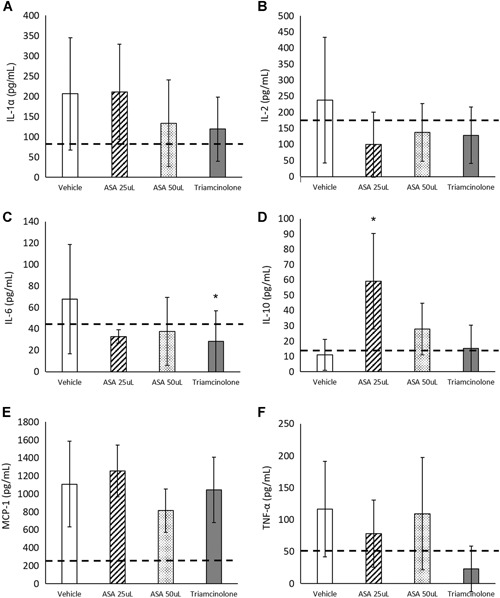
Rat synovial fluid inflammatory panel results for (A) interleukin‐1α (IL‐1α), (B) IL‐2, (C) IL‐6, (D) IL‐10, (E) monocyte chemoattractant protein‐1 (MCP‐1), and (F) tumor necrosis factor‐α (TNF‐α). Mean ± standard deviation reported, with *n* = 10 for all figures. Dashed line represents naïve animals (control). *p < 0.05 compared with the vehicle control by Tukey's post hoc test

Following sacrifice, serum and synovial fluid were collected from rats and analyzed using ELISAs. In serum analyses, there were no significant differences between any of the groups, indicating treatment was localized to the joint and there were no measured systemic effects of ASA (Table 3). Synovial fluid was analyzed for levels of IFN‐γ, IL‐1α, IL‐1β, IL‐2, IL‐4, IL‐6, IL‐10, IL‐13, MCP‐1, and TNF‐α using a multiplex array. IL‐1α, IL‐2, IL‐6, IL‐10, MCP‐1, and TNF‐α had detectable levels (Fig. 4), while the remaining targets were all below the lower limit of detection (data not shown). In this study, IL‐6 levels following ASA treatment were decreased compared with the vehicle control animals (not significant), while treatment with triamcinolone resulted in a significant decrease in IL‐6 levels (*p* < 0.05, Fig. 4C). Interestingly, treatment with 25 μl of ASA resulted in a significant increase in anti‐inflammatory molecule IL‐10 compared with the vehicle controls (*p* < 0.05, Fig. 4D).

**Table 3:** Serum Cytokine Results for IL‐1β, IL‐6, IL‐10, MCP‐1, TIMP‐1, and TNF‐α

Cytokine	Vehicle	ASA (25 μl)	ASA (50 μl)	Triamcinolone
IL‐1β	59.36 ± 7.23	53.79 ± 3.24	57.68 ± 4.85	57.64 ± 5.90
IL‐6	196.6 ± 21.53	204.0 ± 12.98	181.4 ± 42.2	197.0 ± 15.67
IL‐10	209.2 ± 11.92	198.2 ± 27.1	219.6 ± 9.03	228.8 ± 9.12
MCP‐1	1621.7 ± 398.53	1524.59 ± 283.41	1754.15 ± 460.05	1507.56 ± 326.85
TIMP‐1	15133 ± 5231.15	13062 ± 3679.61	13069 ± 4946.56	13205 ± 4333.91
TNF‐α	19.75 ± 0.67	19.52 ± 0.94	19.96 ± 0.67	20.04 ± 0.95

Mean ± standard deviation reported.

IL‐1β, interleukin‐1β; MCP‐1, monocyte chemoattractant protein‐1; TIMP‐1, tissue inhibitors of metalloproteinases; TNF‐α, tumor necrosis factor‐α.

## DISCUSSION

In the MIA rat OA model, treatment with ASA resulted in significant improvement in pain threshold levels and a significant reduction in weight‐bearing difference and swelling. Histological analysis indicated no significant changes in total joint scores between groups; however, the 50 μl ASA and triamcinolone groups resulted in some differences in sub‐scores compared with the vehicle control. Analysis of serum levels showed no systemic effects of ASA treatment, while synovial fluid analysis revealed a significant increase in IL‐10 levels following 25 μl ASA treatment.

Currently, there are limited treatment options that slow or prevent the progression of OA. Common treatments for early‐stage OA range from exercise and physical therapy^31^, ^32^ to pharmacological options such as non‐steroidal anti‐inflammatory drugs (NSAIDs) and supplements (glucosamine, chondroitin sulfate).^32^ Treatments for moderate OA are more limited, and most options are thought to be relatively short‐term solutions. For example, many patients undergo multiple rounds of injections of corticosteroids; while this treatment is often effective at pain relief for 1–6 weeks,^33^ more than 3–4 injections per year are not usually clinically advisable due to concerns over potential degenerative effects on cartilage and surrounding tissue. McAlindon et al.^34^ found significantly greater cartilage volume loss compared with saline following 2 years of IA injections every 12 weeks. In addition, there are several commercially available single or multiple injection hyaluronic acid (HA) formulations. These injections are hypothesized to provide lubrication, mild anti‐inflammatory effects, and pain relief. Meta‐analyses evaluating HA have shown variable responses, and there is increasing concern over a perceived lack of efficacy of some preparations (e.g., low‐molecular weight, non‐crosslinked).^35^ If non‐surgical approaches are not successful, treatment options for advanced OA are limited to partial or total knee arthroplasty, while focal chondral defects can be treated with joint preserving options, including marrow stimulation with or without augmentation, osteochondral autografts and allografts, cell‐based therapy, scaffolds, osteotomy, and meniscal transplantation.^32^, ^36^, ^37^ In addition to traditional OA treatments, advanced biologic options have recently been used clinically with some success including platelet‐rich plasma,^38^ adipose‐derived stem cells,^39^ bone marrow‐derived cells,^40^ allogenic mesenchymal stem cells,^41^ and autologous protein solution (APS).^42^ Furthermore, a pilot clinical study evaluating the safety profile of ASA was recently published by Vines et al.^43^ confirming the safety of ASA for injection and suggesting a clinical benefit for up to 1 year based on patient‐reported outcomes. In the current study, ASA was evaluated as a potential treatment to mitigate the pro‐inflammatory environment of OA by measuring pain relief following treatment with ASA in the MIA rat OA pain model.

Mechanistic data in the MIA model of OA presented here, coupled with early clinical data,^43^ suggest a potential role for ASA as a treatment for OA. Currently, there are limited preclinical studies examining the use of amniotic tissue for OA. In a rat medial meniscus transection (MMT) model, which results in degeneration leading to OA,^44^ micronized amnion and chorion matrix (μ‐dHACM) injection remained in the joint space for at least 21 days.^45^ In addition, MMT models have shown that rats receiving placental‐derived tissues had reduced lesions,^45^, ^46^ cartilage surface erosions,^45^ and increased cartilage thickness and volume.^46^ In a previous clinical pilot study,^43^ no increases in synovitis or fibrosis were observed; however, histological scoring indicated increased inflammation and fibrosis following treatment with ASA compared with the vehicle control, which was seen in another preclinical study using placental‐derived products.^45^

Another interesting finding in this study was increased subchondral bone sclerosis observed with triamcinolone treatment; this finding has been observed in previous in vivo and clinical studies.^47^, ^48^ In this study, behavioral data suggests that triamcinolone exhibits an immediate effect, with significant differences compared with the vehicle control at 8–14 days, while ASA treatment shows a significant effect at 14 days that persists at 21 days. Correspondingly, previous studies have shown that corticosteroids provide a short‐term benefit (1–3 weeks), but taper off, requiring subsequent injections to maintain their effect.^34^, ^48^ This observation may provide insight into the mechanism of how placental‐derived products can improve pain threshold and function scores in patients with knee OA observed in a previous clinical study.^43^

The current study is, to our knowledge, the only study to evaluate pain and function resulting from use of a placental tissue product. Ferreira‐Gomes et al.^30^ showed that treatment of MIA‐induced OA in rats with lidocaine, morphine, and diclofenac, a NSAID, resulted in changes in gait, suggesting pain relief that mimicked the pain relief profile seen in humans. In the current study, ASA treatment resulted in no observed adverse effects, significant improvements in pain threshold and weight bearing differences, and improvements in knee swelling compared with controls. In other MIA rat studies, treatment with coenzyme Q10 (CoQ10) and Celecoxib (a cyclooxygenase‐2 [COX‐2] inhibitor, NSAID) showed significant increases in the pain threshold measured by Von Frey assessment.^49^ To our knowledge, no other studies using an MIA model have shown a reduction in swelling. The reduction in swelling with administration of ASA shows that not only does the injection itself not cause swelling of the knee joint, but also suggests that ASA attenuates swelling associated with inflammation characteristic of the MIA model. In the current study, synovial fluid analyses showed reduced levels of IL‐6 and significantly increased levels of IL‐10 as a result of ASA treatment. Studies evaluating the role of IL‐6 have shown that IL‐6 knockout mice with collagen and antigen‐induced arthritis are protected from joint degradation and inflammation, despite the presence of IL‐1β and TNF‐α in the synovium.^50^ Previously using the MIA model, administration of IL‐10 was shown to provide a chondroprotective effect.^51^ Furthermore, the daily use of oral glucosamine, a common therapy for OA, was shown to increase IL‐10 levels in rats 2 months following MIA induction.^51^ In addition to upregulation of IL‐10 seen in this study, amniotic tissue has also been reported to contain IL‐10.^24^ This in vivo study shows that ASA resulted in improved pain thresholds, decreased weight‐bearing differences and swelling, no systemic changes to serum cytokine levels, and the promotion of anti‐inflammatory environment within the joint space.

The study presented here focused on pain and inflammation in a MIA model of arthritis and evaluated behavioral effects mechanistically. Our study demonstrated changes to the cytokine levels within the joint, improved pain thresholds, and reductions in swelling and weight‐bearing differences. One limitation to this study is that only male rats were used; this decision was made to increase reproducibility within the study between animals and improve chances of seeing differences in treatment due to the known effects of sex and reproductive status on disease severity in OA animal models.^52^, ^53^

Other areas of interest for future work include ASA effects on cartilage degeneration in vivo and the influence of macrophages in vitro and in vivo. Finally, clinical studies validating these effects are necessary; as such, a 200‐patient multicenter study evaluating ASA as a treatment for knee OA has completed enrollment at the time of this evaluation (NCT02318511).

## AUTHORS’ CONTRIBUTION

K.A.K. designed the study, executed experiments, analyzed and interpreted the data, and wrote the manuscript. K.C.M. designed the study, interpreted the data, and wrote and edited the manuscript. A.H.G. and J.F. interpreted the data and wrote and edited the manuscript. All authors have read and approved the final manuscript.
